# Small RNAs: The Essential Regulators in Plant Thermotolerance

**DOI:** 10.3389/fpls.2021.726762

**Published:** 2021-09-17

**Authors:** Zhi-Fang Zuo, Wenbo He, Jing Li, Beixin Mo, Lin Liu

**Affiliations:** ^1^Guangdong Provincial Key Laboratory for Plant Epigenetics, College of Life Sciences and Oceanography, Longhua Bioindustry and Innovation Research Institute, Shenzhen University, Shenzhen, China; ^2^Key Laboratory of Optoelectronic Devices and Systems of Ministry of Education and Guangdong Province, College of Optoelectronic Engineering, Shenzhen University, Shenzhen, China

**Keywords:** microRNA, small interfering RNA, heat stress response, regulatory mechanism, thermotolerance

## Abstract

Small RNAs (sRNAs) are a class of non-coding RNAs that consist of 21–24 nucleotides. They have been extensively investigated as critical regulators in a variety of biological processes in plants. sRNAs include two major classes: microRNAs (miRNAs) and small interfering RNAs (siRNAs), which differ in their biogenesis and functional pathways. Due to global warming, high-temperature stress has become one of the primary causes for crop loss worldwide. Recent studies have shown that sRNAs are involved in heat stress responses in plants and play essential roles in high-temperature acclimation. Genome-wide studies for heat-responsive sRNAs have been conducted in many plant species using high-throughput sequencing. The roles for these sRNAs in heat stress response were also unraveled subsequently in model plants and crops. Exploring how sRNAs regulate gene expression and their regulatory mechanisms will broaden our understanding of sRNAs in thermal stress responses of plant. Here, we highlight the roles of currently known miRNAs and siRNAs in heat stress responses and acclimation of plants. We also discuss the regulatory mechanisms of sRNAs and their targets that are responsive to heat stress, which will provide powerful molecular biological resources for engineering crops with improved thermotolerance.

## Introduction

As sessile organisms, plants are constantly exposed to a wide range of biotic and abiotic stresses that are unfavorable for their growth and development. Abiotic stresses, such as drought, salt, temperature, and heavy metals seriously impact the productivity of plants (Zhao et al., 2016; Zhu, 2016). Due to global warming, high-temperature stress has become one of the primary causes for crop loss (Liu et al., 2017a). Global yields of maize and wheat declined by 3.8 and 5.5%, respectively, due to temperature increases of approximately 0.13°C per decade since 1980 (Lobell et al., 2011). Consequently, the mechanisms for heat stress responses in plants have become a global concern and have received much attention.

Plants have evolved complex and diverse mechanisms to defend against ambient high-temperature stress and various factors are involved in plant thermotolerance, such as heat shock proteins (HSPs), reactive oxygen species (ROS)-scavenging enzymes, heat shock transcription factor (HSFs), and small RNAs (sRNAs; Ohama et al., 2017; Zhao et al., 2021). sRNAs are a class of non-coding RNAs that consist of 21–24 nucleotides (nt) and are critical regulators of gene expression by causing either transcriptional gene silencing (TGS) or post-transcriptional gene silencing (PTGS; Axtell, 2013; D’Ario et al., 2017; Yu et al., 2019). Recently, sRNAs have been reported to participate in heat stress responses and play important roles in plant thermotolerance (Ruiz-Ferrer and Voinnet, 2009; Khraiwesh et al., 2012; Shriram et al., 2016; Liu et al., 2017a; Pagano et al., 2021). In this review, we focus on the roles and the regulatory mechanisms of sRNAs, mainly microRNAs (miRNAs) and small interfering RNAs (siRNAs) underlying heat stress tolerance in plants.

## Small RNAs in Plants

Endogenous sRNAs in plants are classified into two major types based on the tertiary subdivision by Axtell (2013): hairpin RNAs (hpRNAs) and siRNAs. Both hpRNAs and siRNAs result from cleavage of a double-stranded duplex from the helical region of larger RNA precursors by Dicer-like (DCL) enzyme (Axtell, 2013). hpRNAs are derived from a single-stranded RNA (ssRNA) precursor with a stem-loop hairpin structure, whereas siRNAs are derived from a double-stranded RNA (dsRNA) precursors. miRNAs are a well-studied subset of hpRNAs. siRNAs can be divided into two major subgroups including heterochromatic siRNAs (hc-siRNAs) and phased siRNAs (phasiRNAs; Axtell, 2013; Yu et al., 2019). Furthermore, trans-acting siRNAs (tasiRNAs) are a particular class of phasiRNAs that silence targets in trans. All these sRNAs differ in their biogenesis and modes of action.

### MicroRNAs

**Figure 1:**
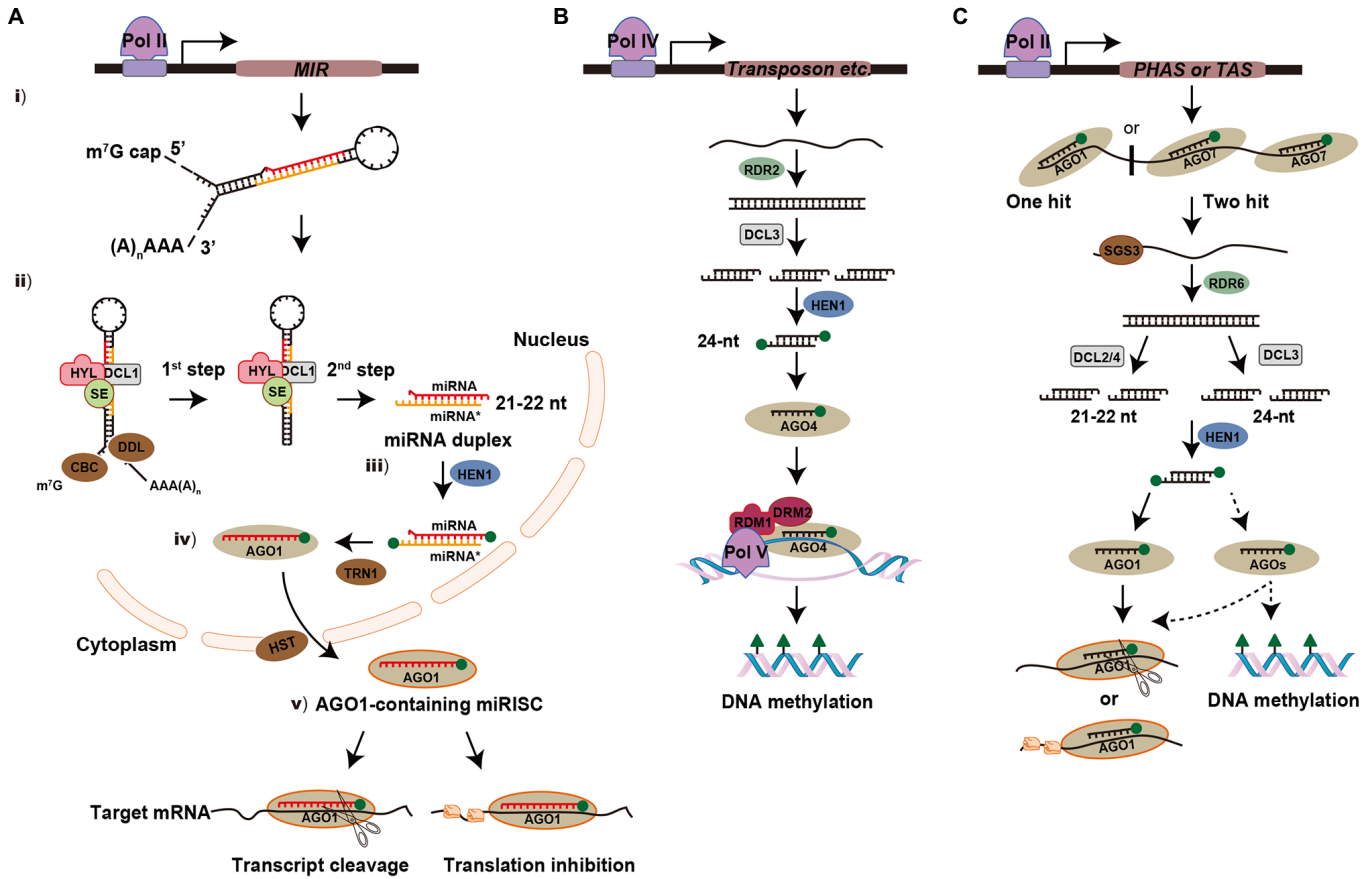
Biogenesis and modes of action of microRNAs (miRNA) and small interfering RNAs (siRNA) in plants. **(A)** Overview of miRNA pathway. *MIR* gene is transcribed by polymerase II (Pol II) to generate a long single-stranded primary miRNA (pri-miRNA) and form a stem-loop or hairpin secondary structure. The pri-miRNA is firstly cleaved into stem-loop precursor miRNA (pre-miRNA) and then processed into miRNA-miRNA* duplex. In this complex process, DCL1 forms a D-bodies with HYPONASTIC LEAVES1 (HYL1) and SE to ensure the accurate and efficient splicing of pre-mRNA. Other cofactors are involved for proper processing, such as cap-binding complex (CBC) and Dawdle (DDL). The miRNA-miRNA* duplex is then methylated by HUA ENHANCER1 (HEN1) to protect the 3' ends from uridylation. The guide strand (miRNA) is incorporated into Argonaute-1 (AGO1) with the aid of TRANSPORIN 1 (TRN1) and then exported from the nucleus to the cytoplasm *via* HASTY (HST). In the cytoplasm, mature miRNA directs post-transcriptional gene silencing (PTGS) *via* transcript cleavage or translational repression. The green balls at the ends of miRNA-miRNA* duplex represent 2'-*O*-methy groups. **(B)** Overview of the heterochromatic siRNA (hc-siRNA) pathway. hc-siRNAs are derived from transposable and repetitive elements. The biogenesis of hc-siRNA requires Polymerase IV (Pol IV) to transcribe a single-stranded RNA (ssRNA) and synthesis into a double-stranded RNA (dsRNA) by RDR2. The dsRNA is then processed into 24-nt siRNA by DCL3. After the siRNA is incorporated into AGO4, Pol V transcribes transcripts that base pairing with siRNA and the siRNA-AGO4 complex is recruited. AGO4, RNA-DIRECTED DNA METHYLATION 1 (RDM1) and DOMAINS REARRANGED METHYLTRANSFERASE 2 (DRM2) trigger DNA methylation at the transcriptional gene silencing (TGS) level. **(C)** Overview of the phased siRNA (phasiRNA) pathway. phasiRNAs originated from phasiRNA-generating (*PHAS*) or trans-acting siRNA (*TAS*) loci are transcribed by Pol II and their precursors are cleaved *via* the miRNA “one-hit” system (AGO1-miRNA complex) or the “two-hit” system (AGO7-miRNA complex). SUPPRESSOR OF GENE SILENCING3 (SGS3) stabilizes the 5'- or 3'-cleaved fragments to protect them from AGO-mediated slicing. Then the primary siRNAs are converted into dsRNAs by RDR6 and further processed into 21- or 22-nt siRNAs by DCL2 and DCL4 or 24-nt siRNAs by DCL3. For further processing, some of them will be recruited by the AGO1 complex and some of them may be incorporated into other uncertain AGOs to trigger the cleavage of target mRNA, translational repression or DNA methylation.

The biogenesis and processing of miRNAs occur in multiple steps in plants (Figure 1A). (i) Similar to protein-coding genes, miRNA-encoded *MIR* genes are transcribed by RNA polymerase II (Pol II) to generate a long single-stranded primary miRNA (pri-miRNA), which is capped and polyadenylated in its 5' and 3' terminal regions, respectively (Lee et al., 2004; Xie et al., 2005). The pri-miRNA is predicted to form a stem-loop or hairpin secondary structure and the imperfectly paired double-stranded stem region contains the miRNA and miRNA* (Meyers et al., 2010). (ii) The pri-miRNA is first cleaved into a stem-loop miRNA precursor miRNA (pre-miRNA) and then the pre-miRNA is processed into miRNA-miRNA* duplex (Schauer et al., 2002; Kurihara and Watanabe, 2004). In this complex process, the RNase III enzyme DCL1 forms a nuclear dicing bodies (D-bodies) in the nucleus with two other partner proteins, HYPONASTIC LEAVES1 (HYL1) and SERRATE (SE), which ensure accurate and efficient splicing of pre-miRNA, resulting in the base of the stem being sliced (Yu et al., 2020). Other cofactors are involved for proper processing of pri-miRNA, such as the Cap-binding complex (CBC; Laubinger et al., 2008) and the Forkhead-associated (FHA) domain-containing protein Dawdle (DDL; Yu et al., 2008). The pre-miRNA without the base is then processed into a miRNA-miRNA* duplex by DCL1, which removes the hairpin loop. (iii) The miRNA-miRNA* duplex is further methylated by methyltransferase HUA ENHANCER1 (HEN1) to protect the 3' ends from uridylation (Li et al., 2005; Yu et al., 2005). (iv) In most cases, the methylated guide strand (miRNA) is incorporated into Argonaute-1 (AGO1) with the aid of TRANSPORIN 1 (TRN1); while the passenger strand (miRNA*) of the duplex is degraded. The AGO1-miRNA complex is exported from the nucleus to the cytoplasm *via* HASTY (HST; Bologna et al., 2018; Yu et al., 2019). (v) The mature miRNA associated with the miRNA-induced silencing complex (miRISC) is guided to target mRNAs for mRNA cleavage by AGO1 (transcript cleavage) or *via* inhibition of protein synthesis (translational repression; Llave et al., 2002; Iki et al., 2010; Li et al., 2013). In addition, the exonucleases SMALL RNA DEGRADING NUCLEASE1 (SDN1), nucleotidyl transferase HEN1 SUPPRESSOR1 (HESO1), and UTP: RNA URIDYLYLTRANSFERASE 1 (URT1) play critical roles in the process of miRNA turnover to regulate its steady-state level (Ramachandran and Chen, 2008; Zhao et al., 2012; Tu et al., 2015).

### Small Interfering RNAs

#### Heterochromatic siRNAs

Heterochromatic siRNAs (hc-siRNAs) are derived from transposable and repetitive elements and are involved in DNA methylation or chromatin alteration by the canonical RNA-directed DNA methylation (RdDM) pathway (Matzke and Mosher, 2014; Borges and Martienssen, 2015). The biogenesis of hc-siRNAs requires RNA Polymerase IV (Pol IV) to transcribe the ssRNA, which is then synthesized into dsRNA by RDR2. The dsRNA is then processed into 24-nt siRNAs by DCL3 and methylated at their 3' ends by HEN1. After the siRNAs is incorporated into AGO4, RNA polymerase V (Pol V) transcribes transcripts that are complementary to the siRNA, and the siRNA-AGO4 complex is recruited. AGO4, RNA-DIRECTED DNA METHYLATION 1 (RDM1), and DOMAINS REARRANGED METHYLTRANSFERASE 2 (DRM2) trigger *de novo* DNA methylation at the TGS level at symmetric CG and CHG sites, or asymmetric CHH sites (H stands for C, T, or A; Figure 1B; Matzke and Mosher, 2014; Du et al., 2015).

#### Phased siRNAs

Phased siRNAs (PhasiRNAs) originated from non-coding phasiRNA-generating (*PHAS*) loci in monocots or protein-coding genes in dicots, which are transcribed by Pol II (Deng et al., 2018; Yu et al., 2018). These target precursors are cleaved using a miRNA-mediated cleavage system: either “one-hit” (AGO1-miRNA complex) or “two-hit” (AGO7-miRNA complex) system. SUPPRESSOR OF GENE SILENCING3 (SGS3) stabilizes the primary siRNAs to protect them from AGO-mediated slicing (Yoshikawa et al., 2013). Then the primary siRNAs are converted into dsRNA by RDR6 and processed into 21- or 22-nt secondary siRNAs by DCL4 or DCL2, respectively. The 21-nt tasiRNAs, which originate from non-coding trans-acting siRNA (*TAS*) loci transcripts are recruited by the AGO1 complex to participate in the cleavage of target mRNAs (Adenot et al., 2006; Fukudome et al., 2011). The biogenesis of 21-nt phasiRNAs largely depends on DCL4 in rice; whereas a class of 24-nt phasiRNAs are processed by the DCL3 homolog DCL3b during the reproductive stage (Song et al., 2012; Komiya, 2017). However, little is known about the downstream processing of these phasiRNAs, such as which AGOs they interact with for silencing remains elusive (Figure 1C).

## Roles of Small RNAs in Plant Thermotolerance

Global warming has diverse and profound effects on plant growth and development, and poses a serious threat to the global crop yields. Therefore, the plant response to high-temperature stress and the mechanism underlying plant thermotolerance have become focuses of research (Bita and Gerats, 2013). Recent studies have shown that plant miRNAs and siRNAs act as key regulators in response to high-temperature stress. Genome-wide studies for heat-responsive sRNAs have been conducted in many plant species using high-throughput sequencing and bioinformatics. A series of heat stress-responsive sRNAs have been identified from various plant species, suggesting that these sRNAs have persistent regulatory roles under extreme temperature (Supplementary Table S1).

### miRNAs Involved in Heat Stress Responses

Extensive studies have shown that miRNAs can target genes encoding a diverse range of regulatory proteins, including a large proportion of TFs, suggesting that miRNAs function at the core of gene regulatory networks. One miRNA family usually has multiple target genes and plays versatile roles in several aspects of plant development and stress resistance. Accumulating evidence has shown that miRNAs are involved in plant responses to heat stress, and act as critical factors in coordinating plant development and heat stress resistance.

#### The miR156/miR172 Family

miR156 and its targets, *SQUAMOSA PROMOTER-BINDING PROTEIN-LIKE* (*SPL*) genes are highly conserved in plants, and regulate developmental phase transitions, including juvenile-to-adult and vegetative-to-reproductive transitions (Xu et al., 2016; He et al., 2019; Ma et al., 2020). miR156 is highly expressed in young seedlings and its expression declines when the shoot develops, displaying opposite changes to its targets (Xu et al., 2016). The miR156-*SPL3* module regulates *flowering locus T* (*FT*) expression to control *Arabidopsis* flowering time in response to ambient temperature (16 and 23°C; Lee et al., 2010; Kim et al., 2012). Besides, miR156 was also found to response to high-temperature stress. Stief et al. (2014a) showed that miR156 isoforms were highly induced after recurring heat stress (37 and 44°C) and promoted sustained expression of heat stress responsive genes in *Arabidopsis*, suggesting miR156 was functionally important for heat stress memory. Heat stress memory refers to the maintenance of acquired thermotolerance that plants obtain after heat stress. Heat stress memory is one of the mechanisms for plants survival under recurring heat stress. Plants can withstand high temperature, which are lethal to them in a normal state. This acquired thermotolerance can be maintained several days after returning to non-stress temperatures (Lämke et al., 2016). miR156 mediated repression of *SPL2* and other target genes enhanced and prolonged the heat stress memory, and this process was also regulated by the HSFA2 cascade, which required HEAT STRESS-ASSOCIATED 32 (HSA32) and ROF1 (Stief et al., 2014a,b). In addition, recent studies showed that miR156-*SPL13* mediates heat stress response in alfalfa, and overexpression of soybean miR156b in *Arabidopsis* led to male sterility under heat stress (Matthews et al., 2019; Ding et al., 2021). These studies suggest that the role of the miR156-*SPL* module is conserved in plants and that plant development and heat stress responses are mediated by miRNAs.

In contrast to miR156, miR172 is a positive regulator in juvenile-to-adult developmental transition by targeting *APETALA2* (*AP2*) family genes, such as *AP2*, *TARGET OF EAT 1* (*TOE1*), *TOE2*, *TOE3*, *SCHLAFMUTZE* (*SMZ*), and *SCHNARCHZAPFEN* (*SNZ*; Gahlaut et al., 2018; Ma et al., 2020). However, miR156 is highly expressed in the juvenile phase; whereas miR172 is barely expressed in this stage (Wu et al., 2009). The targets of miR156, *SPL9*, and *SPL10*, directly promote the expression of miR172b, which indicates that miR172 acts downstream of miR156 to promote adult epidermal identity (Wu et al., 2009). miR172 has been reported to function in thermosensory pathway to regulate ambient temperature-responsive flowering under non-stress temperature conditions. The transgenic plants with overexpression of miR172 showed a temperature insensitive early flowering (Lee et al., 2010). Jung et al. (2012) discovered that RNA-binding protein FCA percept temperature fluctuation and promoted the processing of pri-miR172 *via* recognition of RNA motif in the stem-loop during the early stage of thermosensory flowering pathway. In addition, miR172 has also been revealed to response to high-temperature stress. In both rice post-meiosis panicle and safflower leaf tissues, miR172 was observed to be significantly downregulated, whereas its target *AP2* genes were upregulated under heat stress, indicating an important role of miR172-*AP2* module in plant heat stress response (Kouhi et al., 2020; Peng et al., 2020).

#### The miR159/miR319 Family

The miR159 and miR319 families are highly conserved in plants and have a high degree of sequence identity (Palatnik et al., 2019). miR159 targets several members of *GIBBERELLIC ACID MYB* (*GAMYB*) genes and plays important roles in flowering and male fertility. miR319 targets *TEOSINTE BRANCHED/CYCLOIDEA/PCF* (*TCP*) genes and several *MYB* genes to control leaf growth (Palatnik et al., 2019). miR159 was upregulated by heat stress in flowering Chinese cabbage (Ahmed et al., 2019). In contrast, miR159 was downregulated after heat stress in *Triticum aestivum*, and the transgenic rice overexpressing tae-miR159 showed sensitivity to heat stress (Wang et al., 2012). Heat stress caused a significant decrease of miR159 and an increase of its target genes, *CsGAMYB1* and *CsMYB29*-like in cucumber (Li et al., 2016). In addition, ectopic expression of csa-miR159b in *Arabidopsis* decreased heat tolerance by targeting *AtMYB33* (Li et al., 2016). For miR319, overexpression of sha-miR319d increased expression levels of heat stress-responsive genes and conferred heat stress tolerance in transgenic *Solanum lycopersicum* with increased activities of superoxide dismutase (SOD), catalase (CAT), and ascorbate peroxidase (APX; Shi et al., 2019).

#### The miR160/miR393 Family

The miR160 family targets the *AUXIN RESPONSIVE FACTOR* (*ARF*) gene family, which play vital roles in plant growth and development by regulating auxin signaling (Gahlaut et al., 2018). The miR160-*ARF* module is involved in phenotypic plasticity somatic embryo development, leaf development, root formation, and cell differentiation (Lin et al., 2015, 2018). In *Gossypium hirsutum*, overexpression of miR160 caused sensitivity to heat stress *via* suppressing the expression of *ARF10* and *ARF17* and caused anther indehiscence (Ding et al., 2017). Overexpression of a miR160 precursor presented increased thermotolerance, which shared similar phenotype with *arf10*, *arf16*, and *arf17* mutants in *Arabidopsis* (Lin et al., 2018). Furthermore, miR160 also regulates seed germination, hypocotyl, and rachis growth under heat stress (Lin et al., 2018).

As described above, miR160 is related to auxin. miR393 is also involved in auxin-related development in plants by regulating the expression of the auxin receptors (TAARs) including TRANSPORT INHIBITOR RESPONSE1 (TIR1) and AUXIN SIGNALING F-BOX (AFBs). These TAARs can degrade the *AUXIN/INDOLE-3-ACETIC ACID* (*Aux/IAA*) genes and allow specific ARF TFs to active the auxin-responsive genes, which functions in the primary auxin-responsive pathway (Si-Ammour et al., 2011). Overexpression of osa-miR393a in transgenic creeping bentgrass increased heat tolerance by repressing its targets *AsAFB2* and *AsTIR1*, and this enhanced heat stress tolerance was associated with induced expression of *HSPs* (Zhao et al., 2019).

#### The miR398 Family

Heat stress causes the accumulation of ROS. SODs including iron SOD (Fe-SOD), manganese SOD (Mn-SOD), and copper/zinc SOD (Cu/Zn-SOD) encoded by *CSDs* are important ROS-scavenging enzymes that catalyze the superoxide radicals in plants. miR398 family members were found to be rapidly induced by heat stress, leading to the downregulation of their target genes *CSD1*, *CSD2*, and *Copper chaperone for SOD* (*CCS*) in *Arabidopsis* (Guan et al., 2013). Furthermore, *csd1*, *csd2*, and *ccs* mutants showed heat stress tolerance with increased expression of *HSFs* and *HSPs* (Guan et al., 2013). Fang et al. (2019) showed that the induction of tocopherols and 3'-phosphoadenosine 5'-phosphate (PAP) are required for the increased accumulation of miR398 and acquisition of heat tolerance. In addition, HSFA1b and HSFA7b were revealed to be responsible for the heat induction of miR398 by binding directly to the promoter of *MIR398* to activate its transcription (Guan et al., 2013). A recent study further revealed that the *MIR398* genes possess their natural antisense transcripts (NATs) and uncovered a regulatory loop between them; the *cis*-NATs of *MIR398* genes repress the processing of miR398 pri-miRNAs, which cause poorer thermotolerance due to the upregulation of miR398-targeted genes (Li et al., 2020). However, the underlying mechanism of how heat stress regulates the expression of *MIR398 cis*-NATs remains to be investigated.

#### Other miRNA Families

Other development-related miRNAs are also associated with plant heat stress responses. miR169 family members can target *Nuclear transcription factor Y subunit* (*NF-YA*) genes and function at flowering stage in rice under heat stress, which was confirmed by overexpression of miR169r-5p (Liu et al., 2017b). The paradigmatic miR396-*GROWTH-REGULATING FACTOR* (*GRF*) model is well established and plays important roles in regulating the size of multiple plant tissues or organs (Liu et al., 2021). Interestingly, Giacomelli et al. (2012) reported that miR396 mediated the cleavage of *HaWRKY6* in sunflower during early responses to high temperature. Heat stress reduced the accumulation of miR396, which showed opposite expression patterns to *HaWRKY6*, and expression of a miR396-resistant version of *HaWRKY6* altered heat stress responses in *Arabidopsis* (Giacomelli et al., 2012). For *MIR400* family, a heat stress-induced alternative splicing event was observed to occur in the intron of *MIR400*, which was co-transcribed with its host gene in *Arabidopsis* (Yan et al., 2012). Under heat stress, the alternative splicing of the *MIR400* intron resulted in greater accumulation of miR400 primary transcripts and reduced level of mature miR400. In addition, overexpression of miR400 caused higher sensitivity to heat stress in transgenic plants compare to the wild type plants. These results demonstrated that miR400 acts as a negative regulator in plant heat stress resistance and revealed the essential role of alternative splicing in linking miRNA and high-temperature stress (Yan et al., 2012). A report of rice miR5144-3p showed that miR5144-3p plays a role in protein folding and abiotic stress during rice development by regulating the expression of *OsPDIL1;1* (Xia et al., 2018). miR5144-3p was downregulated under heat stress, leading to the increased accumulation of *OsPDIL1;1* mRNA in rice; STTM-miR5144-3p and *OsPDIL1;1* overexpression transgenic rice exhibited improved heat tolerance (Xia et al., 2018).

### siRNAs Involved in Heat Stress Responses

A large number of miRNAs and putative siRNAs participate in plant responses to environmental stresses, such as dehydration, salinity, cold, and ABA (Sunkar and Zhu, 2004). Compared with the large number of miRNAs and siRNAs identified in responses to other environmental stresses in plants, hitherto siRNAs identified in response to heat stress is relatively few. Previous researches have reported that heat-induced copia-type retrotransposon *ONSEN* was accumulated in the siRNAs biogenesis impaired mutants, which revealed the potential roles of siRNAs in plant heat stress response (Ito et al., 2011). The accumulation of a particular class of phasiRNAs-tasiRNAs derived from *Arabidopsis TAS* loci were found to decrease significantly under heat stress, indicating their participation in plant heat stress responses, and their functions in thermotolerance were subsequently investigated (Zhong et al., 2013; Li et al., 2014). Overexpression of *TAS1a*-derived tasiRNAs in *Arabidopsis* downregulated target genes *HEAT-INDUCED TAS1 TARGET1* (*HTT1*) and *HTT2*, and led to weaker thermotolerance in transgenic plants; whereas overexpression of *HTT1* and *HTT2* led to improved thermotolerance *via* upregulation of several *HSFs* (Li et al., 2014). Furthermore, heat-induced tasiRNA decrease was found to be involved in thermomemory of early flowering and attenuated immunity through targeting *HTT5*, which provides insights for understanding how heat exposure influence the fitness of plant progeny (Liu et al., 2019). Hu et al. (2020) have also identified a newly evolved phasiRNA locus that generated consecutive 21-nt phasiRNAs in response to heat stress in *Camellia*. Predictive bioinformatics and gene expression analysis showed that these secondary phasiRNAs could potentially target several genes including *LIPOXYGENASE*, *RAN GTPase*, *XYLOGLUCAN ENDOTRANSGLUCOSYLASE*, and *ATPase* to regulate their expression in a *trans*-acting manner. However, further genetic studies are required to verify these targets and elucidate the specific function of these phasiRNAs in thermal resistance. In a recent study, a large population of transposable element derived 24-nt siRNAs were significantly reduced in maize tassels and roots after exposure to high temperature, and genes nearby these transposable elements tended to be downregulated, indicating that the expression of heat-dependent gene is influenced by adjacent transposon sequences. However, the underlying mechanism controlling the relationship among transposable element, transposable element derived 24-nt siRNAs and nearby genes in response to heat stress remains elusive (He et al., 2019).

**Figure 2:**
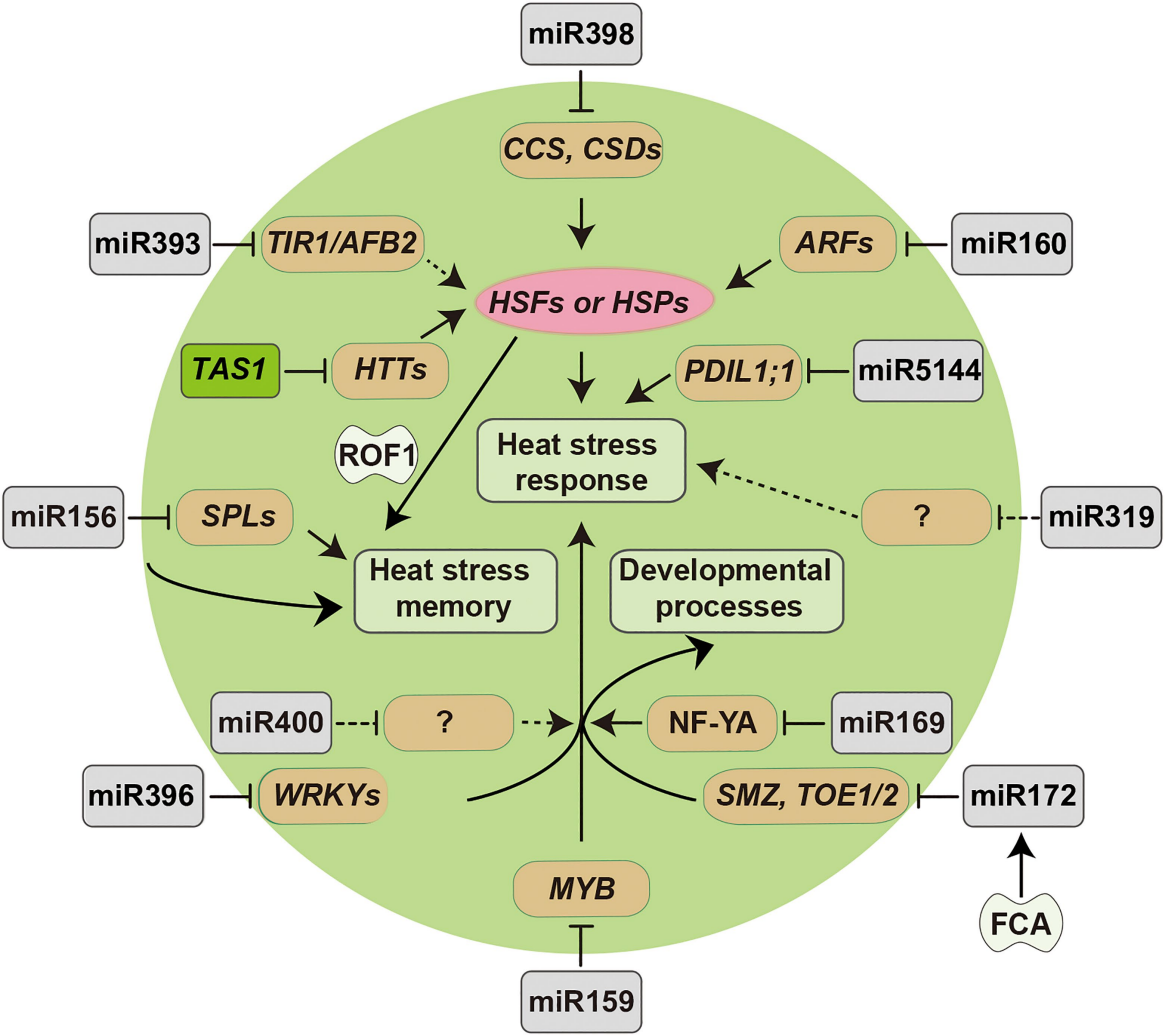
Diagram of miRNA-target modules involved in plant responses to heat stress. Arrowed lines represent promotional effects and flat lines represent repressive effects. Dashed lines indicate hypotheses that need to be confirmed.

The findings above demonstrate that sRNAs can serve as vital regulators of plant responses to heat stress. The major miRNAs and siRNAs involved in heat stress resistance and their regulatory pathway have been summarized in Figure 2. Interestingly, some sRNAs tend to display species-dependent expression patterns under heat stress, indicating that they may have distinct regulatory mechanisms in different plant species. For example, miR397 was downregulated in rice and tomato, but upregulated in banana in response to high temperature stress (Liu et al., 2017b; Pan et al., 2017; Zhu et al., 2019). In addition, several studies have identified novel species-specific sRNAs by genome-wide deep sequencing. For example, Liu et al. (2015b) identified 25 novel heat stress-responsive miRNAs in *Saccharina japonica*, such as sja-novel-mir-5, sja-novel-mir-13, and sja-novel-mir-59. These species-specific sRNAs may have essential roles in plant heat stress responses and dissecting their functions will broaden our understanding of the underlying regulatory mechanisms governing thermotolerance in different plant species.

## Molecular Mechanisms of Plant Responses to High-Temperature Stress

Heat stress causes many adverse effects on plant growth, development, and physiological processes. Reduced water content, excess generation of ROS, and protein denaturation caused by heat stress greatly impede normal cellular functions (Hasanuzzaman et al., 2013; Jacob et al., 2017). Plants adapted to respond to and survive from heat stress by developing diverse mechanisms to cope with severe conditions, such as basal thermotolerance (Bokszczanin and Fragkostefanakis, 2013). Currently, heat stress response pathways have been extensively investigated in plants, and mainly include the HSP-based protective pathway, phospholipid pathway, ROS/redox signaling pathway, and phytohormone signaling pathway (Bokszczanin and Fragkostefanakis, 2013; Qu et al., 2013).

Heat-induced changes in plasma membrane fluidity cause a transient opening of Ca^2+^ channels, which induces Ca^2+^ influx to the cytoplasm. The increased levels of cytosolic Ca^2+^ activate multiple kinases, such as Calcium-dependent protein kinases (CDPKs), thereby evoking the expression of transcriptional regulators in response to heat stress. HSFs and HSPs play critical roles in this process (Qu et al., 2013; Guo et al., 2016). HSFs are evolutionarily grouped into A, B, and C classes. The *HSFA* subfamily genes, *HSFA1*-*HSFA9* have been well studied. *HSFA1s* function as master regulators, which are indispensable in acquired thermotolerance of plants by activating downstream heat stress responsive transcription factors (TFs), such as MULTIPROTEIN BRIDGING FACTOR1C (MBF1C) and DEHYDRATION-RESPONSIVE ELEMENT-BINDING PROTEIN2A (DREB2A; Liu et al., 2011; Yoshida et al., 2011). HSPs, including HSP70, HSP90, HSP100, and HSP101 are the main inducers of HSFs, which resolve large aggregates induced by unfolded proteins and reduce protein misfolding (Jacob et al., 2017). In addition, HSP70 and HSP90 repress the activity of HSFA1s under non-stress condition by protein–protein interactions (Ohama et al., 2017). Phosphorylation and dephosphorylation by CALMODULIN-BINDING PROTEIN KINASE3 (CBK3) and PROTEIN PHOSPHATASE7 (PP7), respectively, can activate HSFA1s *via* post-translational modification under heat stress.

Heat stress causes remodeling of lipids in the membrane, which may induce the phospholipid signaling and the key mediators in this pathway include phosphatidyl inositol 4,5-bisphosphosphate (PIP2) and phosphatidic acid (PA). Accumulation of lipid signaling molecules can in turn trigger Ca^2+^ influx through channels in the plasma membrane (Bokszczanin and Fragkostefanakis, 2013). The mechanisms behind phospholipid signaling, from heat signal initiation to transduction, and the relationship between lipid signaling and plasma membrane channels as yet are still much unknown in plants.

Heat stress induces unfolded or misfolded proteins in the cytosol and endoplasmic reticulum (ER) that may trigger the unfolded protein response (UPR). Cytosolic UPR is mainly associated with specific HSFs, such as HSFA2, which is induced by HSFA1s and regulated by one splice variant of its own, S-HSfA2 *via* post-transcriptional regulation (Sugio et al., 2009; Liu and Charng, 2013). Two signaling pathways are involved in UPR, proteolytic processing, mediated by the basic leucine zipper domain (bZIP) TFs, RNA splicing, mediated by Inositol-requiring enzyme 1 (IRE1; Deng et al., 2013). ROS that accumulates during heat stress responses, such as H_2_O_2_, can act as signaling molecules to trigger the ROS-scavenging pathway; Respiratory burst oxidase homologs (RBOHs) also play important roles for the initiation and signal propagation of this pathway (Baxter et al., 2014). Furthermore, ROS-scavenging antioxidant enzymes, Late embryogenesis abundant (LEA) proteins, osmolytes, and secondary metabolites are considered necessary for detoxification of ROS (Bokszczanin and Fragkostefanakis, 2013).

Phytohormones are key players in plant growth and development from seed germination to senescence, and are involved in plant adaptation to adverse ambient stresses. Strigolactone (SL), cytokinin (CK), abscisic acid (ABA), and ethylene (Et) regulate the leaf senescence under heat stress (Abdelrahman et al., 2017). In addition, salicylic acid (SA) and jasmonic acid (JA), which are responsive to abiotic stresses, are also involved in the regulation of plant *HSF* genes (Guo et al., 2016; Rai et al., 2020). One phytohormone signaling pathway can respond to multiple abiotic stresses *via* crosstalk because plants endure multiple stresses in nature. For example, heat stress is usually associated with high light.

## Mechanisms of Small RNA Regulation in Plant Thermotolerance

### Morphological Acclimation of Plants Under Heat Stress by sRNAs

**Figure 3:**
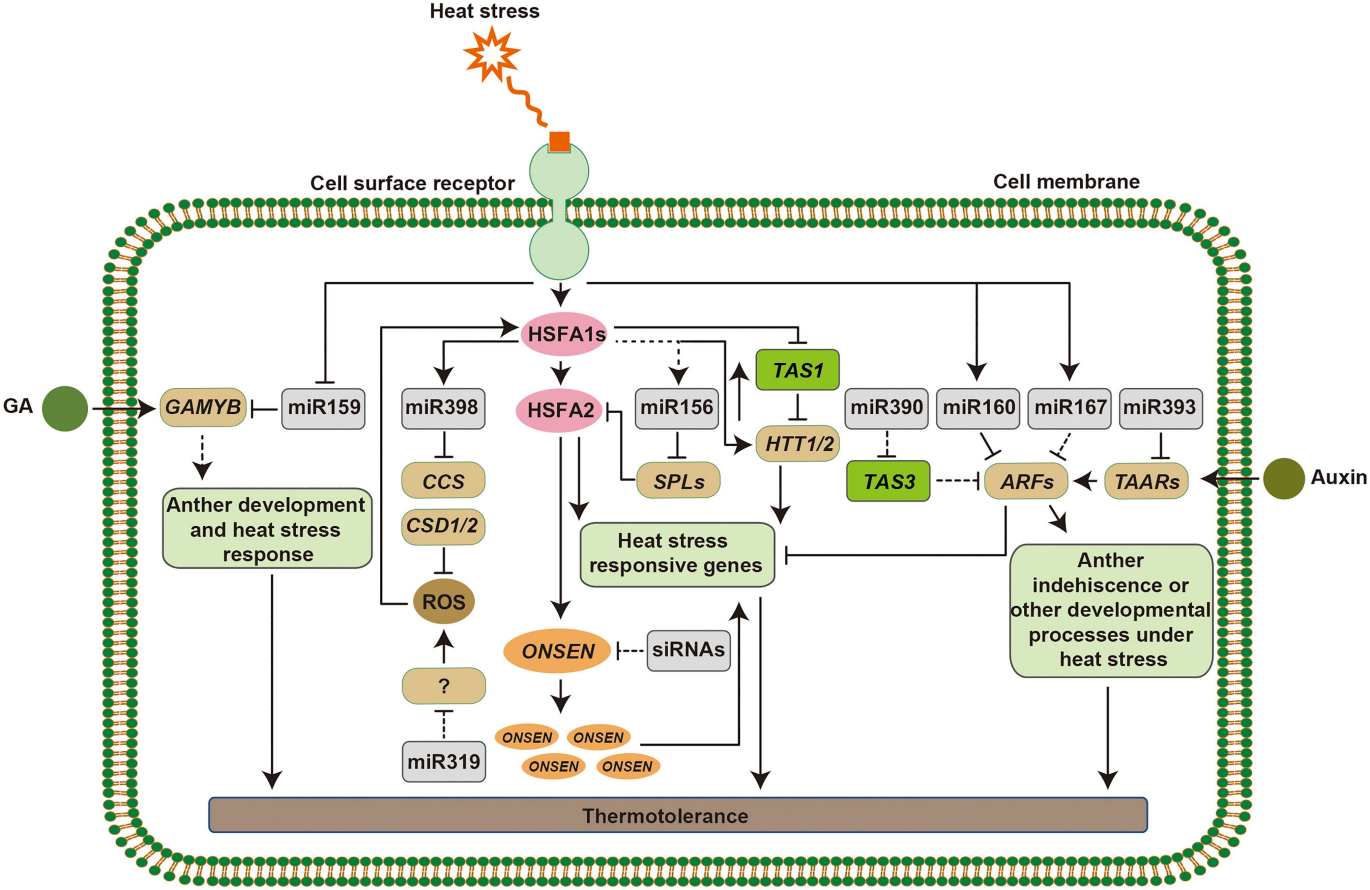
Mechanism of small RNA (sRNA) regulation in plant responses to heat stress. Heat stress signaling can be perceived by putative sensors on the cell membrane, which induce the expression of heat stress responsive genes. HSFA1s function as master regulators to active the downstream heat stress responsive transcription factors and genes. Heat-induced miR398 downregulates *CSDs* and *CCS*. Accumulation of reactive oxygen species (ROS) in the cells acts as signals to alter the expression of heat shock transcription factors (*HSFs*), and miR319 functions in this pathway *via* unknown targets. HSFA1s directly activate the expression of *HTT* genes, targets of *TAS1*, which upregulate *HSF* and heat shock protein (*HSP*) genes. The miR156-*SPL* module promotes sustained expression of heat stress-inducible genes in response to recurring heat stress and prolong the heat stress memory. In addition, the retrotransposition *ONSEN* is activated by HSFAs, although, siRNAs regulate its activity, which can be inherited to its progeny. miR159-*GAMYB* modules function in gibberellin (GA) signaling. miR160, miR167, miR390, and miR393 target *AUXIN RESPONSIVE FACTOR* (*ARF*) or *TAARs* that act in the auxin response pathway and show morphological adaptations under heat stress. Arrowed lines represent promotional effects and flat lines represent repressive effects. Dashed lines indicate hypotheses that need to be confirmed.

Ambient temperature fluctuations affect plant functioning, geographical distribution, and agricultural production of crops (Proveniers and van Zanten, 2013). The phenotypic responses of plants to deal with high temperature include hypocotyl elongation, leaf hyponasty, and floral induction. The warm temperature induced basic helix–loop–helix (bHLH) TF, PHYTOCHROME-INTERACTING FACTOR 4 (PIF4) plays a central role in warmth-mediated morphological acclimation (Franklin et al., 2011; Kumar et al., 2012; Kim et al., 2020). miRNAs are also involved in morphological adaptations of plant under heat stress. For example, overexpression of miR160 improved seed germination and increased the length of hypocotyl elongation and the rachis (Lin et al., 2018). The reproductive phase of flowering plants is highly sensitive to high temperature, which often contributes to the acceleration of flowering and results in poor seed set (Zinn et al., 2010). As indicated above, the heat-responsive miR156, miR159, miR172, and miR319 regulate flowering time or male and female fertility in different plant species, which illustrates that miRNAs play important roles in triggering the development of flower set (Supplementary Table S1; Wu et al., 2009; Lee et al., 2010; Stief et al., 2014a; Yin et al., 2018; Ahmed et al., 2019; Hu et al., 2019; Zhu et al., 2019; Kouhi et al., 2020). Furthermore, the heat-induced retrotransposon *ONSEN* was accumulated during flower development and before gametogenesis in mutants that are deficient in siRNA synthesis (Ito et al., 2011). Taken together, it is suggested that sRNAs play pivotal roles in regulating plant growth and reproductive tissue development under heat stress, which ensures transgenerational seed production (Figure 3).

### Regulation of Essential Factors of HSFs/HSPs in Heat Stress Responses by sRNAs

Heat shock proteins that act as molecular chaperones are major functional proteins in heat stress response *via* the activation of HSFs (Qu et al., 2013; Ohama et al., 2017). Various attempts have been made to increase thermotolerance in different host by overexpression of a single *HSF* or *HSP* gene, such as *CaHSP25.9*, *ZmHSF05*, *TaHSP23.9*, and *OsHSP20*, (Feng et al., 2019; Li et al., 2019; Guo et al., 2020; Wang et al., 2020). Nevertheless, *AsHSP26.8a*, a novel chloroplast-localized small *HSP* gene from creeping bentgrass negatively regulates heat stress resistance through modulating ABA and other stress signaling pathways (Sun et al., 2020). In addition, several miRNAs affect heat stress responses by targeting and activating *HSF/HSP* genes. The miR156-*SPL* module downregulates the expression of heat stress inducible genes, such as *HEAT STRESS ASSOCIATED 32* (*HSA32*), *HSP17.6A*, and *HSP22.0* during recovery from heat stress, which is functionally important for heat stress memory (Stief et al., 2014a). Overexpression of miR160 altered the expression of *HSPs* including *HSP17.6A*, *HSP17.6II*, *HSP21*, and *HSP70B*, which allow plants to survive under heat stress (Lin et al., 2018). In *Solanum habrochaites*, constitutive expression of sha-miR319d enhanced heat tolerance *via* upregulation of *HSFA1a* and *HSFA1b*, while overexpression of Osa-miR393a caused higher expression levels of *AsHSP17.0* and *AsHSP26.7a* than in wild type plants (Shi et al., 2019; Zhao et al., 2019). Transgenic plants expressing miR398-resistant forms of *CSD1*, *CSD2*, or *CCS* showed reduced expression levels of *HSF* genes (*HSFA1e*, *HSFA2*, *HSFA3*, and *HSFA7b*) and *HSP* genes (*HSP17.6*, *HSP70B*, and *HSP90.1*), while *csd1*, *csd2*, and *ccs* loss-of-function mutants showed enhanced thermotolerance with increased expression of heat stress inducible genes (Guan et al., 2013). Furthermore, HSFA1b and HSFA7b bind to heat stress elements directly in the promoter region of miR398b, which constituted a positive regulatory feedback loop (Figure 3). Overexpression of *HTT1* and *HTT2*, targets of *TAS1*, upregulated several *HSF* genes and enhanced thermotolerance in *Arabidopsis* (Li et al., 2014). Meanwhile, HSFA1a directly activates the expression of *HTT* genes, such as *HTT1*, which act as cofactors of HSP70-14 complexes in the thermotolerance pathway (Li et al., 2014). All of these discoveries reveal that sRNAs help plants achieve thermotolerance by regulating the expression of *HSFs* and *HSPs* (Figure 3).

### sRNAs Mediated Heat Stress Responses Trigger the ROS-Scavenging Pathway

Unfavorable environments trigger the production of ROS, which causes oxidative damages to proteins, lipids and stress-induced electrolyte leakage in plants. Plants have evolved an antioxidant defense system equipped with various enzymatic and non-enzymatic ROS-scavengers to maintain an equilibrium between the production of ROS and elimination of excessive ROS (Caverzan et al., 2019). Several antioxidant enzymes, such as SOD, APX, and CAT are involved in the heat stress responses in the ROS-scavenging pathway, where oxidative stress is produced as a secondary stress (Qu et al., 2013). As described above, downregulation of *CSD1*, *CSD2*, and *CCS* by heat-induced miR398 led to accumulation ROS in cells, which contributed to the expression of *HSFs* and other heat stress-responsive genes (Guan et al., 2013). This regulatory mechanism constitutes a regulatory loop for plant thermotolerance that involves miR398, *HSF* genes, and ROS-scavenging enzymes. A recent study showed that, tocopherols and PAP positively regulated the biogenesis of miR398 in chloroplasts and promoted plant thermotolerance (Fang et al., 2019). In addition, altered ROS under heat stress may act as signals in cells to induce the expression of *HSF1Aa* and *HSF1Ab* in miR319d transgenic plants, but the mechanisms of ROS, miR319d, and its putative targets in this signal transduction are much unknown (Shi et al., 2019; Figure 3). These findings suggest that sRNAs-mediated heat stress responses are partially dependent on the ROS signaling pathways in plants.

### Heat Stress Responsive sRNAs Involved in Phytohormone Signaling

Phytohormones are produced *via* environmental signals, such as heat stress. For example, miR159-regulated GAMYB-like TFs function in gibberellin (GA) signaling and overexpression of tae-miR159 caused sensitivity to heat stress in transgenic plants, which suggested that tae-miR159 may participate in a heat stress related signaling pathway (Murray et al., 2003; Wang et al., 2012). Auxin orchestrates many morphogenetic processes, such as root formation and anther development, and endogenous auxin is also involved in heat stress responses. miR160 and miR393 target *ARFs* or *TAARs*, which are components in the auxin response pathway (Si-Ammour et al., 2011; Kruszka et al., 2014; Gahlaut et al., 2018). Overexpression of miR160 in cotton increased sensitivity to heat stress and caused anther indehiscence by activating the auxin response; while constitutive expression of miR157 suppressed the auxin signal, which also caused sensitivity to heat stress with microspore abortion and anther indehiscence (Ding et al., 2017). Overexpressing of an miR160 precursor in *Arabidopsis* significantly reduced the expression of its target genes *ARF10*, *ARF16*, and *ARF17*, and led to improved tolerance of transgenic plants. Furthermore, *arf10*, *arf16*, and *arf17* mutants showed advanced thermotolerance by regulating the expression of *HSPs* (Lin et al., 2018). In addition, the miR167-*ARFs and* miR390-*TAS3-ARFs* models are involved in plants developmental processes; however, whether miR167 and miR390 function in heat stress response by targeting *ARFs* need to be tested (Figure 3). ABA induces the accumulation of miR168, and both plants overexpressing miR168a and loss-of-function mutant of its target *AGO1*, ago1-27, displayed ABA hypersensitivity and several abiotic stress tolerances (Li et al., 2012). miR168 also responds to heat stress in various species, such as *Arabidopsis*, rice, *Brassica rapa*, and flowering Chinese cabbage (Barciszewska-Pacak et al., 2015; Bilichak et al., 2015; Mangrauthia et al., 2017; Ahmed et al., 2019). Responses of plants to heat stress are complex, and may require physiological or metabolic changes from several phytohormone signaling pathways with a crosstalk, and there is no doubt that sRNAs are essential regulators in these processes.

### sRNAs Are Involved in Heat Stress Memory

As discussed above, a multi-layered regulatory signaling pathways are involved in plant response to heat stress. Heat stress memory as one of the mechanisms for plants survival under recurring heat stress included the regulation of chromatin modifications. For example, high levels of histone H3 lysine 4 (H3K4) methylation are related to hyper-induction of heat stress inducible genes after a recurring heat stress, which depends on HSFA2 (Yoshida et al., 2011; Liu and Charng, 2013; Lämke et al., 2016). miR156 promotes sustained expression of heat stress inducible genes in response to recurring heat stress, and increases of miR156 prolong heat stress memory (Stief et al., 2014a,b). miR156 targets, *SPLs*, are critical for heat stress memory, and may serve to integrate morphological acclimation with heat stress responses. Furthermore, plants display transgenerational memory mediated by transposons. Transgenerational memory refers to transmitting epigenetic states or environmental responses from one generation to the next that may offer the offspring an adaptive advantage for better fitness (Liu et al., 2015a). A surprisingly high frequency of new heat-induced retrotransposition, *ONSEN* insertions in progeny after heat stress revealed that the transgenerational memory of heat stress is maintained during differentiation of generative organs by priming *ONSEN* to transpose (Ito et al., 2011). In addition, the activation of *ONSEN* requires heat-induced TFs in the heat stress response pathway, such as HSFA2 (Cavrak et al., 2014). Although, the mechanisms of DNA methylation, sRNAs, and transposons in heat stress memory are obscure, epigenetic regulation is an important mechanism in response to heat stress (Figure 3).

## Conclusion and Perspectives

Heat stress caused by the global warming affects the growth and development of plants, which increases the risk of yield reductions in agricultural crops. It is important to elucidate how plants respond to heat stress, but many questions remain to be answered, such as how do plants sense heat stress, and what kinds of signaling pathways the sensors use to transduce the signals into nucleus? Given that epigenetic regulation by sRNAs is crucial in gene regulatory networks for heat stress responses, we believe that an intensive understanding of their roles and functions will provide plentiful potential biological resources for plant engineering. Recently, various heat stress responsive sRNAs have been identified. However, it should be noted that many experimental factors affect the responses of sRNAs in plants, such as plant species, tissue, developmental stage, treatment time, and growth conditions, which means that a slight change can lead to different expression patterns of sRNAs. We summarized the literatures on sRNAs involved in heat stress responses of plants and listed the heat stress conditions used for treatments in Supplementary Table S1. Even though, a large number of diverse heat stress responsive sRNAs have been identified in plants, their roles and molecular mechanisms are still not fully elucidated, which may be due to the lack of genetic materials, especially for non-model plants and important crops. Future research should focus on the creation of genetically modified mutants and genetic manipulations of sRNAs to identify additional sRNA-target modules in the regulatory networks of heat stress responses. Notably, most miRNAs that have been functionally investigated so far are evolutionary conserved miRNAs, whereas the species- or tissue-specific miRNAs have been rarely studied. Thus, much more work is required to decipher the regulatory mechanisms of non-conserved miRNAs in response to heat stress in more crops. In addition, the investigation of upstream regulation of heat-responsive miRNA would also be an interesting research topic and worthy of more attention.

As discussed above, several sRNAs were shown to have potential in improving plant thermotalerance. One type of sRNAs is involved in the heat stress responses by targeting and activating HSF/HSP genes that act as molecular chaperones to prevent denaturation or aggregation of target proteins, such as miR160 and miR393 (Lin et al., 2018; Zhao et al., 2019). Thus, increasing the saturation level of HSFs and HSPs by overexpression of one specific miRNA can enhance heat tolerance to their host plants using transgenic approaches. The other type of sRNAs participate in heat stress response by triggering ROS-scavenging pathway in plants, such as miR319 and miR398 (Guan et al., 2013; Shi et al., 2019). High levels of ROS-scavenging enzymes accumulation by manipulating certain miRNAs can be another strategy to generate heat stress tolerant plants. Recently, new biotechnological tools have been successfully explored to investigate *MIR* genes or miRNA modulation, such as endogenous artificial target mimicry (Short tandem target mimicry, STTM), miRNA transient virus induced gene silencing (VIGS) and *MIR* genes editing using CRISPR/Cas9 system (Basso et al., 2019). Furthermore, next-generation sequencing, for example, sRNA sequencing (sRNA-seq), Parallel Analysis of RNA Ends (PARE) analysis (Zhai et al., 2014; Jiang et al., 2020), and recently developed single-cell sRNA-mRNA co-sequencing (Wang et al., 2019) have provided powerful methods for elucidating the functions of sRNAs and their target genes. These techniques will be essential in further research and will expand the range of sRNA applications for crop breeding in thermotolerance.

Ultimately, the gene-silencing mechanisms mediated by sRNAs explore a new vista in the application of genetic engineering, which can be used to revolutionize agriculture by controlling a wide array of crop traits, including thermotolerance. sRNAs can work efficiently and precisely to develop targeted gene-silencing approaches in plants for various requirements, which can be used not only for the study of the functional analysis of genes responsive to heat stress but also to improve crop plants by manipulating their target genes. All of these accumulated researches will enable the successful and extensive application of sRNA technology for the development of next generation crops.

## Author Contributions

Z-FZ and LL wrote the manuscript. WH, JL, and BM provided the critical comments and edited the manuscript. All authors contributed to the article and approved the submitted version.

## Funding

This work was supported by Shenzhen Grant Plan for Science and Technology (JCYJ20190808112207542), Guangdong Innovation Research Team Fund (2014ZT05S078), Natural Science Foundation of Guangdong Province (2019A1515011222 and 2021A1515010482), and Guangdong Basic and Applied Basic Research Foundation (2019A1515110162).

## Conflict of Interest

The authors declare that the research was conducted in the absence of any commercial or financial relationships that could be construed as a potential conflict of interest.

## Publisher’s Note

All claims expressed in this article are solely those of the authors and do not necessarily represent those of their affiliated organizations, or those of the publisher, the editors and the reviewers. Any product that may be evaluated in this article, or claim that may be made by its manufacturer, is not guaranteed or endorsed by the publisher.
